# Orthostatic Systolic Blood Pressure Elevation and Incident Atrial Fibrillation: Insights From the SPRINT Trial

**DOI:** 10.1111/jch.70122

**Published:** 2025-08-22

**Authors:** Jue Wang, Wenhe Lv, Zhen Wang, Sitong Li, Zhixian Wang, Le Zhou, Yufeng Wang, Lan Ren, Chao Jiang, Liu He, Shijun Xia, Xiangyi Kong, Song Zuo, Yu Kong, Xueyuan Guo, Xiaoxia Liu, Songnan Li, Ribo Tang, Deyong Long, Caihua Sang, Ning Zhou, Xin Du, Jianzeng Dong, Changsheng Ma

**Affiliations:** ^1^ Department of Cardiology Beijing Anzhen Hospital Capital Medical University and National Clinical Research Center for Cardiovascular Diseases Beijing China; ^2^ Heart Health Research Center Beijing China; ^3^ Department of Cardiology The First Affiliated Hospital of Zhengzhou University Zhengzhou Henan Province China

**Keywords:** atrial fibrillation, blood pressure variability, hypertension, orthostatic, systolic blood pressure

## Abstract

Exaggerated orthostatic changes in systolic blood pressure (SBP) were associated with adverse cardiovascular events. We aim to assess the association between orthostatic SBP changes and incident atrial fibrillation (AF). We performed a post hoc analysis of SPRINT (Systolic Blood Pressure Intervention Trial). Orthostatic SBP changes were defined as standing SBP minus seated SBP. Patients were grouped into tertiles of orthostatic SBP changes. We used Cox proportional regression models to assess the association of orthostatic SBP changes with incident AF. Among 8455 participants included in this analysis, 327 incident AF cases occurred during follow‐up. After adjusting for age, female, race, smoking, alcohol use, history of cardiovascular disease, history of chronic kidney disease, and body mass index, an SBP increase ≥6 mmHg to standing was independently associated with a 43% higher risk of incident AF (HR: 1.43; 95% CI: 1.07–1.90; *p* = 0.014) compared to nonsignificant orthostatic SBP changes (>–4 to <6 mmHg). A SBP decrease ≥4 mmHg to standing showed a nonsignificant higher risk of developing AF compared to SBP changes of >–4 to <6 mmHg. In subgroup analysis, the results presented a similar tendency to the main result. Sensitivity analyses also generated consistent results while additionally adjusting for seated and standing blood pressure or heart rate. In this post hoc analysis of the SPRINT trial, exaggerated SBP increase on standing independently predicts incident AF.

**Trial Registration**: ClinicalTrials.gov identifier: NCT00000620.

## 1 Introduction

Atrial fibrillation (AF) is the most common clinical arrhythmia, leading to a higher risk of heart failure, stroke, and death [1]. Among adults aged over 40 years, hypertension is the main modifiable risk factor for AF, with a population‐attributable risk of 15.9%–22.4% [2]. In patients with hypertension, positional variability in blood pressure is a marker of derangement of autonomic nervous system adaptation to postural changes, associated with adverse cardiovascular events [3, 4]. In recent years, the prognostic role of orthostatic hypertension has been accumulating [5]. Observational studies demonstrated that, orthostatic increase in systolic blood pressure (SBP) ≥20 mmHg was associated with a higher risk of heart failure, cardiovascular mortality, and mortality in older population [6, 7]. In a young hypertensive cohort, an SBP increase of >6.5 mmHg from lying to standing independently predicted major adverse cardiovascular and renal events [8]. However, the relationship between excessive orthostatic changes in SBP and AF development remains unclear.

In this post hoc analysis of SPRINT (Systolic Blood Pressure Intervention Trial) [9], we aim to examine the prospective association between orthostatic SBP changes and risk of incident AF in patients with hypertension.

## 2 Methods

### 2.1 Study Population

The SPRINT trial was a multicenter, open‐label, randomized controlled trial that compared the effects of an intensive SBP target of <120 mmHg with a standard SBP target of <140 mmHg in patients with hypertension. The design and results of the SPRINT trial have been previously published [9, 10].

SPRINT included 9361 participants whose SBP was between 130 and 180 mmHg at the screening with increased cardiovascular disease risks. They excluded those with diabetes mellitus, prior stroke, or a standing SBP <110 mmHg. All participants from SPRINT with seated and standing SBP measurements at baseline were enrolled in this analysis. We excluded participants with missing or uninterpretable baseline ECG, those without any follow‐up ECG, as well as patients with preexisting AF (Figure S1).

### 2.2 Blood Pressure Measurement

The seated blood pressure and heart rate were obtained as the mean of three measurements in the clinic, using an appropriate cuff size with an Omron oscillometric device (Omron Healthcare, Inc., Bannockburn, IL). The standing blood pressure and heart rate were the first standing measurements taken 1 min after the participants’ feet touched the floor.

Orthostatic changes in SBP were calculated by subtracting the average seated SBP from the single measurement of standing SBP [11]. A positive orthostatic SBP change indicates that the SBP increases in response to standing; a negative value indicates that the SBP decreases upon standing. Orthostatic changes in diastolic blood pressure (DBP) and heart rate were calculated in a similar way, respectively.

### 2.3 AF Ascertainment

Participants having a history of AF or with AF present on baseline ECG were defined as having preexisting AF [12]. Incident AF was defined as the first occurrence of AF ascertained from the scheduled and unscheduled standard 12‐lead ECGs in patients without preexisting AF [12]. Scheduled ECG was obtained at baseline, year 2, year 4, and at a close‐out visit in the SPRINT trial. Unscheduled ECG took place when participants were suspected of serious adverse events by the SPRINT investigators.

Digital ECG data were recorded at 10 mm/mV calibration and a speed of 25 mm/s by a GE MAC 1200 electrocardiograph (GE, Milwaukee, WI). ECG readings were performed at the Epidemiological Cardiology Research Center, Wake Forest School of Medicine (Winston‐Salem, NC). All ECG tracings were initially inspected visually for technical errors and inadequate quality, and then automatically processed using GE 12‐SL Marquette version 2001. Additionally, all ECG tracings which were classified as AF and those with Minnesota ECG Classification codes that suppress the detection of AF were confirmed by SPRINT ECG Reading Center staff.

### 2.4 Statistical Analysis

Baseline characteristics were compared by tertiles of orthostatic SBP changes using descriptive statistics. Continuous variables were described as mean ± SD or median (25th, 75th percentiles) and compared using the analysis of variance test or Kruskal–Wallis test. Categorical variables were summarized as counts and percentages and compared using chi‐square test.

The distribution of orthostatic SBP changes was visualized using histograms. Kaplan–Meier curves were generated for cumulative incidence of incident AF by orthostatic SBP changes tertiles and compared using log‐rank test. The Cox proportional hazards model was used to assess the associations between orthostatic SBP changes groups and incident AF. No violations to the proportional hazards assumption were detected by inspection of the Schoenfeld residuals. Age, sex, and race, smoking, alcohol use, history of cardiovascular disease, history of chronic kidney disease, and body mass index (BMI) were fitted in the Cox model. Seated and standing blood pressure and heart rate were additionally adjusted for models in the sensitivity analysis. We also used restricted cubic splines to assess the nonlinear association between orthostatic SBP changes and incident AF. Models with four knots at the 5th, 35th, 65th, and 90th percentiles of orthostatic SBP changes were displayed.

We assessed the effect modification of the association between orthostatic SBP change and incident AF across age (<75 vs. ≥75 years), sex, prior cardiovascular disease, prior chronic kidney disease, SBP tertiles (≤132, >132 to <145, ≥145 mmHg), and treatment arms. Follow‐up time was censored at the date of the last ECG or death. A 2‐tailed *p* value <0.05 was considered statistically significant. All analyses were performed using R version 4.2.2.

## 3 Results

### 3.1 Study Population Characteristics

The mean age of the 8455 participants was 67 ± 9 years, 3052 (36.1%) were female, and 1568 (18.5%) had a history of cardiovascular disease. At baseline, the mean blood pressure of the population was 140 ± 16/78 ± 12 mmHg in the seated position and 140 ± 18/82 ± 13 mmHg on standing. The average seated‐to‐standing blood pressure difference was 1 ± 12/4 ± 8 mmHg (Table 1). The participants were categorized into tertiles: orthostatic SBP changes of ≤–4, >–4 to <6, and ≥6 mmHg. Compared with participants who had seated‐to‐standing SBP changes between >–4 and <6 mmHg, both those with orthostatic SBP changes ≤–4 mmHg and those with SBP changes ≥6 mmHg were more likely to be older, female, white, and had higher BMI (Table 1).

**TABLE 1:** Baseline characteristics.

		Orthostatic SBP changes			
Characteristics	Total (*N* = 8455)	≤–4 mmHg (*n* = 2863)	>–4 to <6 mmHg (*n* = 2769)	≥6 mmHg (*n* = 2823)	*p* value
Age, years	67.5 (9.3)	68.4 (9.4)^a^, ^c^	66.7 (9.2)^a^, ^b^	67.4 (9.3)^b^, ^c^	<0.001
Female	3052 (36.1)	1105 (38.6)^a^	916 (33.1)^a^, ^b^	1031 (36.5)^b^	<0.001
White	5404 (63.9)	1939 (67.7)^a^, ^c^	1758 (63.5)^a^, ^b^	1707 (60.5)^b^, ^c^	<0.001
Smoking	1227 (14.5)	457 (16.0)^c^	401 (14.5)	369 (13.1)^c^	0.009
Alcohol	339 (4.0)	121 (4.2)	110 (4.0)	108 (3.8)	0.742
History of CVD	1568 (18.5)	578 (20.2)^a^	457 (16.5)^a^, ^b^	533 (18.9)^b^	0.002
History of CKD	2319 (27.4)	914 (31.9)^a^, ^c^	723 (26.1)^a^	682 (24.2)^c^	<0.001
BMI, kg/m^2^	29.9 (5.8)	29.0 (5.6)^a^, ^c^	29.9 (5.6)^a^, ^b^	30.8 (6.0)^b^, ^c^	<0.001
eGFR, mL/min/1.73 m^2^	72.2 (20.5)	69.9 (20.8)^a^, ^c^	72.9 (20.4)^a^	73.7 (20.2)^c^	<0.001
Intensive arm	4222 (49.9)	1408 (49.2)	1412 (51.0)	1402 (49.7)	0.372
Number of BP‐lowering agents	2.0 [1.0, 3.0]	2.0 [1.0, 3.0]^c^	2.0 [1.0, 2.0]	2.0 [1.0, 2.0]^c^	0.073
Seated‐to‐standing changes					
SBP, mmHg	0.7 (11.8)	−11.8 (7.3)^a^, ^c^	1.0 (2.6)^a^, ^b^	13.0 (6.7)^b^, ^c^	<0.001
DBP, mmHg	3.7 (7.5)	−0.5 (7.0)^a^, ^c^	3.6 (5.9)^a^, ^b^	8.1 (7.1)^b^, ^c^	<0.001
HR, bpm	5.3 (5.1)	5.8 (5.5)^a^, ^c^	5.0 (4.8)^a^	5.1 (4.9)^c^	<0.001

*Note*: Values are mean ± SD, median (interquartile range), or *n* (%).

Abbreviations: BMI, body mass index; BP, blood pressure; CKD, chronic kidney disease; CVD, cardiovascular disease; DBP, diastolic blood pressure; eGFR, estimated glomerular filtration rate; HR, heart rate; LDL, low‐density lipoprotein; SBP = systolic blood pressure.

^a^ *p* < 0.05 for Group 1 (orthostatic SBP changes ≤ ‐4 mmHg) versus Group 2 (orthostatic SBP changes >‐4 to <6 mmHg).

^b^ *p* < 0.05 for Group 2 versus Group 3 (orthostatic SBP changes ≥ 6 mmHg).

^c^ *p* < 0.05 for Group 1 versus Group 3.

### 3.2 Association of Orthostatic Blood Pressure Changes and Incident AF

**FIGURE 1:**
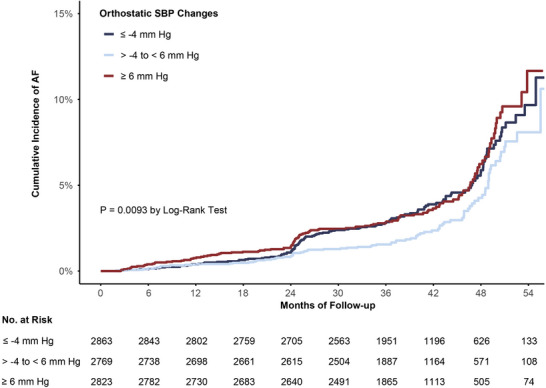
Incidence of incident atrial fibrillation stratified by tertiles of orthostatic changes in systolic blood pressure. SBP, systolic blood pressure.

During a mean follow‐up of 3.9 years (32 363 person‐years), there were 327 cases of incident AF. The incidence rate of AF development was 12.0 (95% CI 10.7–13.3) per 1000‐person years. The Kaplan–Meier for survival free from AF in the three groups was depicted in Figure 1.

The univariate and multivariate Cox regression analyses were subsequently performed to assess the association between the three orthostatic SBP response groups and incident AF. In the unadjusted model, exaggerated orthostatic SBP changes of ≤–4 and ≥6 mmHg were both associated with a higher risk of incident AF (Table 2). In the multivariate models, compared to the SBP changes of –4–6 mmHg, the risk of developing AF was not statistically significant in the SBP changes of ≤–4 mmHg group (Table 2). Adjustment for demographic characteristics (Model 1) and additional cardiovascular risk factors (Model 2) did not substantially alter the association between SBP increase of ≥6 mmHg and the higher risk of incident AF. In Model 1 and Model 2, the hazard ratios (HRs) were 1.48 (95% CI: 1.12–1.96, *p* = 0.006) and 1.43 (95% CI: 1.07–1.90, *p* = 0.014), respectively (Table 2).

**TABLE 2:** Estimated associations between orthostatic change in systolic blood pressure and incident atrial fibrillation.

Orthostatic SBP changes	≤‐4 mmHg		>‐4 to <6 mmHg	≥6 mmHg	
Events/Participants	124/2863		84/2769	119/2823	
IR* (95% CI)	13.3 (11.1–15.8)		9.3 (7.4–11.5)	13.2 (11.1–15.8)	

Hazard ratio	HR (95% CI)	*p* value		HR (95% CI)	*p* value
Unadjusted model	1.44 (1.09‐1.90)	0.011	Ref.	1.50 (1.13‐1.99)	0.004
Model 1	1.26 (0.95‐1.67)	0.104	Ref.	1.48 (1.12‐1.96)	0.006
Model 2	1.23 (0.93‐1.63)	0.150	Ref.	1.43 (1.07‐1.90)	0.014

*Note*: IR^*^, per 1000 person‐years. Model 1 was adjusted for age, sex, and race. Model 2 was additionally adjusted for smoking, alcohol use, history of cardiovascular disease, history of chronic kidney disease, and body mass index.

Abbreviations: HR, hazard ratio; IR, incidence rate; SBP, systolic blood pressure.

**FIGURE 2:**
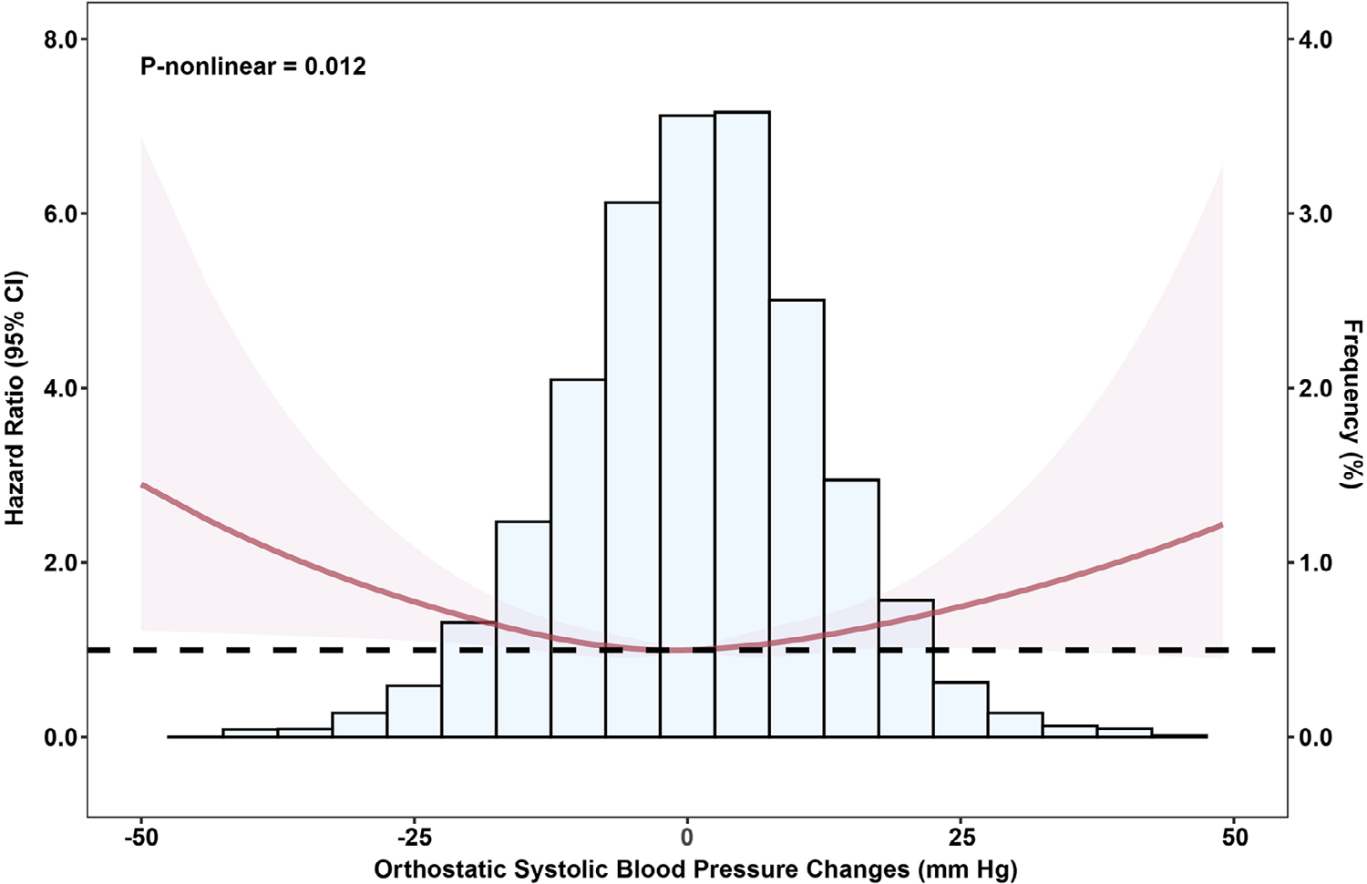
Restricted cubic splines of orthostatic changes in systolic blood pressure to estimate atrial fibrillation development risk in the unadjusted model. The orthostatic SBP changes were determined by subtracting the seated SBP from the standing SBP measurements. SBP, systolic blood pressure.

We further assessed the orthostatic SBP change continuously using restricted cubic regression splines. The U‐shaped association was observed between orthostatic SBP changes and incident AF in the unadjusted model (P for nonlinearity = 0.012) (Figure 2), and the relationship was not significant in the adjusted model (P for nonlinearity = 0.239) (Figure S2).

### 3.3 Subgroup and Sensitivity Analyses

**FIGURE 3:**
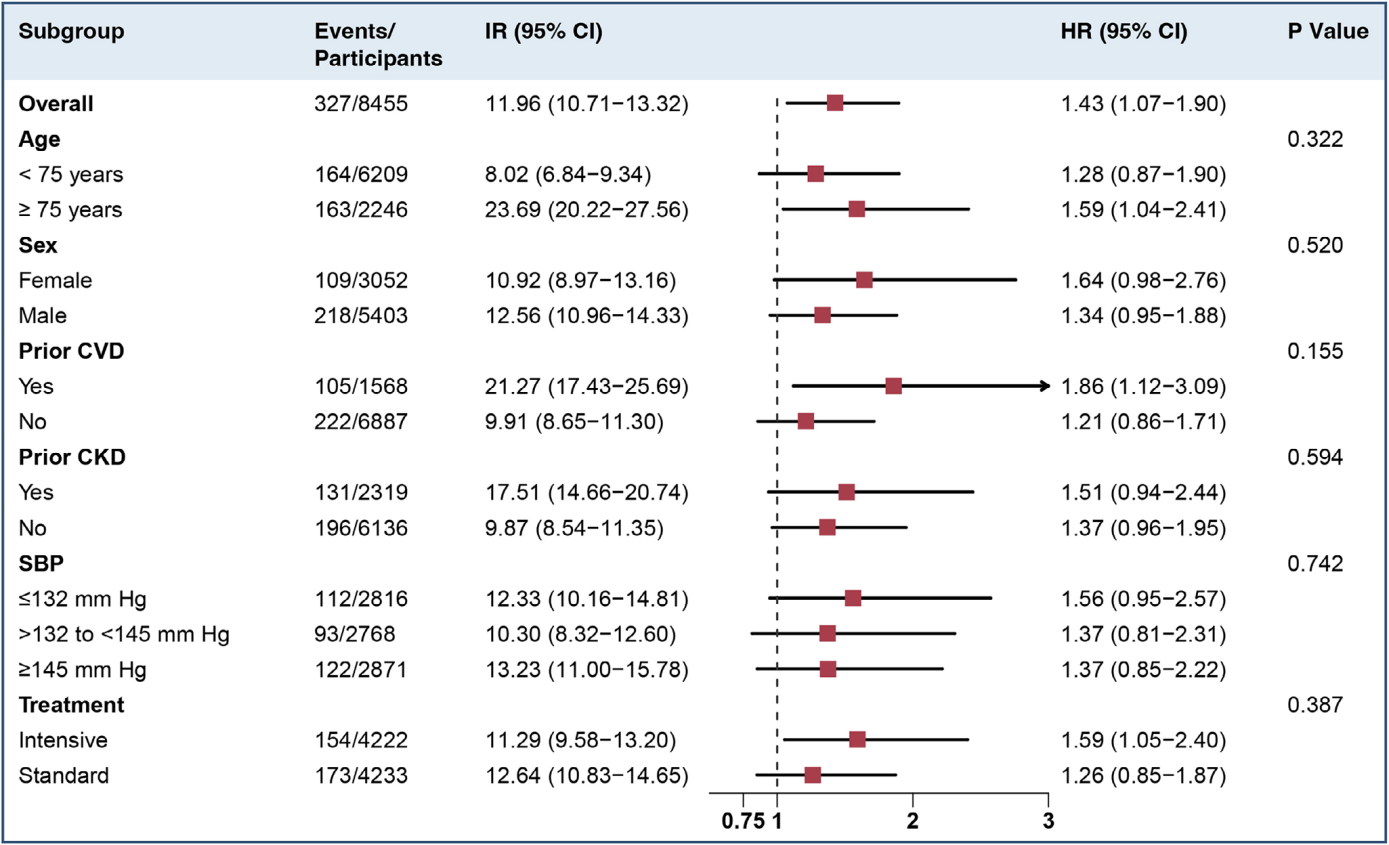
Subgroup analysis of hazard ratios for incident atrial fibrillation in patients with exaggerated orthostatic SBP increase and normal changes. IR per 1000 person‐years. The forest plot represents the HRs for the incident atrial fibrillation in patients with an exaggerated SBP increase of ≥6 mmHg, using normal orthostatic SBP changes of >–4 to <6 mmHg as the reference group. Models were adjusted for age, sex, race, smoking, alcohol use, history of cardiovascular disease, history of chronic kidney disease, and body mass index. HR, hazard ratio; IR, incidence rate; SBP, systolic blood pressure.

There was no evidence of effect modification of the association between exaggerated seated‐to‐standing SBP increase and incident AF by age, sex, history of cardiovascular disease or chronic kidney disease, baseline SBP levels, and SBP target (Figure 3).

When further adjusted for baseline seated SBP and DBP, the association between an SBP change of ≥6 mmHg and incident AF remained significant (Table S1). Similarly, when adjusting for standing blood pressure, the association between an SBP change of ≥6 mmHg and AF risk was also statistically significant (Table S2). The result remained consistent when further adjusting for seated and standing heart rate (Table S3).

## 4 Discussion

In this post hoc analysis of the SPRINT trial, an orthostatic SBP increase of ≥6 mmHg was significantly associated with a 43% higher risk of developing AF, compared with individuals with modest or no SBP changes (–4–6 mmHg). Although a statistically significant association was not observed in those with a marked SBP reduction of ≤–4 mmHg, a 23% increased AF risk was noted. Our study highlights the association between exaggerated orthostatic SBP responses and incident AF and extends the current evidence about the prognostic role of orthostatic SBP elevation and adverse cardiovascular outcomes.

The definition of orthostatic hypertension remained unsettled in the literature. The American Autonomic Society and the Japanese Society of Hypertension defined it as an orthostatic increase in SBP of at least 20 mmHg with an SBP of at least 140 mmHg while standing [13]. In recent years, the clinical significance of exaggerated orthostatic increase has also grown with the accumulation of evidence. Smaller SBP increases in response to standing have also shown clinical relevance. An analysis from the CARDIA study found that an SBP increase >5 mmHg upon standing was associated with a higher risk of hypertension, with an 8‐year incidence of 12.4%, compared to 6.8% and 8.4% in those with SBP changes of –5–5 or >5 mmHg decrease [14]. The HARVEST study followed 1207 stage 1 hypertensive patients between 18 and 45 years for 17.2 years, showing that an SBP increase >6.5 mmHg upon standing was associated with a higher risk of cardiovascular and renal events (HR 1.97, 95% CI 1.10–3.52), including stroke, myocardial infarction, revascularization, heart failure hospitalization, permanent AF, and chronic kidney disease [8]. However, no studies have explored the relationship between orthostatic hyperreactivity in blood pressure and incident AF. Our study provides the population‐based evidence for this association.

Blood pressure is a dynamic physiologic variable, presenting an integrative response to environmental stimulations and physiological conditions. During postural transitions, nearly 500 mL of blood rapidly shift away from chest to the venous system below the diaphragm. To maintain cardiovascular homeostasis, compensatory mechanisms including neural, humoral, vascular, renal, and rheological pathways, are activated [4]. Mainly regulated by baroreceptor reflex, and to a lesser extent by chemoreceptor reflex, the sympathetic nervous system stimulates, leading to increased heart rate, myocardial contractility, and peripheral vasoconstriction [15]. Additionally, the local veno‐arteriolar axon reflex reacts and increases vascular resistance upon standing [15]. These responses stabilize blood pressure within 1 min or less. Prolonged upright further reduces central volume via transcapillary shift, with mechanoreceptors and the renin‐angiotensin‐aldosterone system ongoing regulation [16]. Ultimately, similar or slightly smaller changes in systolic and diastolic blood pressure were observed during the upright changes in normal subjects [17].

Dysfunction in any of the aforementioned mechanisms may lead to inappropriate blood pressure responses during orthostasis. However, direct pathophysiological evidence underlying orthostatic exaggerated blood pressure increase remained limited. Observational studies suggest that the potential mechanisms may differ across age groups. In younger individuals, orthostatic hypertension appears to be associated with excessive neurohumoral activation and impaired baroreceptor reflex, resulting in increased sympathetic nervous activity and inordinate constriction of small arteries. One early study demonstrated that, in patients with orthostatic hypertension, postural changes led to increased pooling of blood in the lower extremities, followed by elevated plasma norepinephrine levels and excessive constriction of peripheral resistance arterioles after 5–60 min of standing [18]. Similarly, in the HARVEST study, orthostatic hyperreactors exhibited higher 24‐h urinary norepinephrine excretion and elevated sympathetic tone [19]. In elderly adults, arterial stiffness appears to play a prominent role in this condition. In an elderly cohort with a mean age of 88 years, patients with orthostatic hypertension showed only a 1.5 bpm increase in heart rate upon standing compared to normotensive individuals [7]. The researchers suggested that the presented adrenergic response was insufficient to explain the blood pressure changes, and therefore, arterial stiffness and reduced cardiac preload upon standing might be predominant contributors to orthostatic hypertension in elderly patients [7, 20].

One of the potential associations between orthostatic hypertension and AF may be mediated by autonomic dysfunction. The atria are innervated by the sympathetic and parasympathetic nerves [21]. The incoordinate activity of sympathetic and parasympathetic nerves is one of the precipitating causes of atrial arrhythmias. Sympathetic stimulation shortens the abbreviated action potential within the pulmonary vein, facilitating early afterdepolarizations and triggering rapid firing within the pulmonary vein [22]. Non‐homogeneous vagal overactivity across the atrial wall, shortens part of the action potential and refractory period, leading to intra‐atrial re‐entry [23]. Additional mechanisms include adrenergically mediated Ca^2+^ loading, L‐type Ca^2+^ channels opening, aberrant Ca^2+^ releases, and delayed afterdepolarization formation [24]. Arterial stiffness, another potential contributor to excessive orthostatic hypertension, is independently associated with AF. Shared risk factors, such as diabetes, chronic kidney disease, obesity, and aging, promote AF through endothelial dysfunction, inflammation, and cardiac remodeling [25]. In addition, genome‐wide association studies and Mendelian randomization have further supported this association [26, 27].

Although observational and experimental studies have suggested potential mechanisms, direct causal evidence linking exaggerated orthostatic blood pressure increase to AF development remains lacking. Further studies are required to establish this relationship.

In this study, a 23% increased risk of developing AF was also observed in patients with greater SBP reductions after standing, although this association did not reach statistical significance. Orthostatic hypotension predicts adverse cardiovascular outcomes and all‐cause mortality [28]. Results from an observational cohort study of 33 346 individuals with 24 years of follow‐up indicated that orthostatic hypotension was associated with a 44% higher risk of new‐onset AF in hypertensive patients [29]. The potential pathophysiological link between orthostatic hypotension and AF includes several aspects [30]. Higher blood pressure variability may provoke intermittent bouts of increased afterload, leading to end‐organ damage. Furthermore, orthostatic hypotension is a common manifestation of autonomic dysfunction [30]. Exaggerated orthostatic SBP reduction may trigger the activation of neuroendocrine compensatory mechanisms and promote AF development [31]. SPRINT excluded individuals with one‐minute standing SBP <110 mmHg, a population particularly susceptible to orthostatic hypotension. The exclusion of these high‐risk individuals may have reduced the statistical power to detect the significant association between orthostatic SBP reductions and incident AF.

Based on the potential mechanistic interrelation, orthostatic SBP elevation may act as a marker indicating the involvement of autonomic dysfunction or atrial stiffness in the triggering and maintaining mechanisms of AF. Therapeutic strategies upstream targets of AF may represent a better approach to prevent the incidence and recurrence of AF.

### 4.1 Limitations

Several limitations should be acknowledged in this study. First, the SPRINT trial scheduled an ECG every 2 years during follow‐up and at study completion, which allowed for AF ascertainment. It is possible that some asymptomatic or paroxysmal AF failed to be detected due to intermittent monitoring. Thus, we also included AF cases diagnosed in the reporting of serious adverse events to minimize missed cases. Nevertheless, the true incidence of AF is likely to have been underestimated. This limitation is particularly relevant to this study of orthostatic SBP changes, as abnormal autonomic modulation, which is a key underlying mechanism, is also a common trigger of paroxysmal AF [32]. The potential under‐detection of asymptomatic or paroxysmal AF may have attenuated the observed association between exaggerated orthostatic SBP changes and AF risk. This effect may be particularly pronounced in patients with orthostatic SBP reductions of ≤–4 mmHg, potentially diluting the strength of the association.

Second, orthostatic blood pressure changes are time‐dependent and reflect a dynamic physiological response to postural transition. Previous studies have reported varying definitions based on measurements taken at 1, 3, or 5 min after standing or during tilt testing from supine or seated positions. Based on the temporal variability of orthostatic blood pressure responses, the 2024 ESC guidelines recommend at least two blood pressure measurements at 1 and 3 min after standing to define orthostatic hypotension [33]. Similarly, in patients with paroxysmal AF, tilt testing has demonstrated that orthostatic SBP elevations also follow a dynamic pattern [34]. Thus, a single standing BP reading at 1 min may therefore be insufficient to capture the full trajectory of the orthostatic response. In this study, due to protocol constraints in SRRINT, standing SBP was recorded only at 1 min after standing from a seated position. This methodological limitation may have resulted in incomplete characterization of orthostatic SBP changes, and the identified threshold in this study may not be generalized to other definitions based on different timings or postural protocols.

## 5 Conclusions

In hypertensive patients with high cardiovascular risk, exaggerated SBP increase on standing independently predicts incident AF compared with relatively stable changes responding to upright posture. Orthostatic SBP changes may act as a marker indicating the potential upstream mechanisms inducing AF.

## Author Contributions

J.W. and Z.W. conceptualized the manuscript. J.W. and W.L. wrote the original draft, processed, analyzed, visualized, and interpreted the data. Z.W. and Y.W. contributed to data acquisition. C.J., X.D., and C.M. critically revised the manuscript. All authors approved the final version of the manuscript.

## Disclosure

Dr Changsheng Ma has received honoraria for presentations from AstraZeneca, Bayer Healthcare, Boehringer Ingelheim, Bristol‐Myers Squibb, Johnson & Johnson, and Pfizer. Dr Jianzeng Dong has received honoraria for presentations from Johnson & Johnson. All other authors declare no competing interests.

## Ethics Statement

This study was approved by the ethics committees of Beijing Anzhen Hospital, Capital Medical University. The procedures used in this study adhere to the Declaration of Helsinki.

## Consent

This study used data from the SPRINT. The protocol was approved by each Institutional Review Board and all participants provided informed consent.

## Conflicts of Interest

The authors have no financial conflicts of interest.

## Supporting information




**Table S1**: Estimated associations between orthostatic change in systolic blood pressure and incident atrial fibrillation additionally adjusted for seated SBP and DBP.
**Table S2**: Estimated associations between orthostatic change in systolic blood pressure and incident atrial fibrillation additionally adjusted for standing SBP and DBP.
**Table S3**: Estimated associations between orthostatic change in systolic blood pressure and incident atrial fibrillation additionally adjusted for seated and standing heart rate.
**Figure S1**: Selection of study population.
**Figure S2**: Restricted cubic splines of orthostatic changes in systolic blood pressure to estimate atrial fibrillation development risk in the adjusted model.

## Data Availability

All SPRINT data for this study are publicly available at the National Heart, Lung, and Blood Institute Biologic Specimen and Data Repository Information Coordinating Center (BioLINCC) and can be accessed from https://biolincc.nhlbi.nih.gov/home/.
