# Supplementary material for: Orthostatic Systolic Blood Pressure Elevation and Incident Atrial Fibrillation: Insights From the SPRINT Trial
Source: J Clin Hypertens (Greenwich). 2025 Aug 22;27(8):e70122. doi: 10.1111/jch.70122 (PMC12373152; doi:10.1111/jch.70122)
Supplement: Supplementary file 1 — Table S1: Estimated associations between orthostatic change in systolic blood pressure and incident atrial fibrillation additionally adjusted for seated SBP and DBP. Table S2: Estimated associations between orthostatic change in systolic blood pressure and incident atrial fibrillation additionally adjusted for standing SBP and DBP. Table S3: Estimated associations between orthostatic change in systolic blood pressure and incident atrial fibrillation additionally adjusted for seated and standing heart rate. Figure S1: Selection of study population. Figure S2: Restricted cubic splines of orthostatic changes in systolic blood pressure to estimate atrial fibrillation development risk in the adjusted model. [file JCH-27-e70122-s001.docx]

**Supplementary Material**

**Table S1. Estimated associations between orthostatic change in systolic blood pressure and incident atrial fibrillation additionally adjusted for seated SBP and DBP**

| **Orthostatic SBP changes** | **HR (95% CI)** | **P value** |
| --- | --- | --- |
| ≤-4 mmHg | 1.23 (0.92-1.63) | 0.160 |
| >-4～<6 mmHg | Ref. |  |
| ≥6 mmHg | 1.42 (1.07-1.89) | 0.017 |

The model was adjusted for age, sex, race, smoking, alcohol use, history of cardiovascular disease, history of chronic kidney disease, body mass index, seated SBP, and steated DBP.

DBP = diastolic blood pressure; SBP = systolic blood pressure.

**Table S2. Estimated associations between orthostatic change in systolic blood pressure and incident atrial fibrillation additionally adjusted for standing SBP and DBP**

| **Orthostatic SBP changes** | **HR (95% CI)** | **P value** |
| --- | --- | --- |
| ≤-4 mmHg | 1.25 (0.93-1.68) | 0.132 |
| >-4～<6 mmHg | Ref. |  |
| ≥6 mmHg | 1.41 (1.05-1.88) | 0.022 |

The model was adjusted for age, sex, race, smoking, alcohol use, history of cardiovascular disease, history of chronic kidney disease, body mass index, standing SBP, and standing DBP.

DBP = diastolic blood pressure; SBP = systolic blood pressure.

**Table S3. Estimated associations between orthostatic change in systolic blood pressure and incident atrial fibrillation additionally adjusted for seated and standing heart rate**

| **Orthostatic SBP changes** | **HR (95% CI)** | **P value** |
| --- | --- | --- |
| ≤-4 mmHg | 1.22 (0.92-1.62) | 0.174 |
| >-4～<6 mmHg | Ref. |  |
| ≥6 mmHg | 1.41 (1.06-1.87) | 0.018 |

The model was adjusted for age, sex, race, smoking, alcohol use, history of cardiovascular disease, history of chronic kidney disease, body mass index, seated heart rate, and standing heart rate.

SBP = systolic blood pressure.


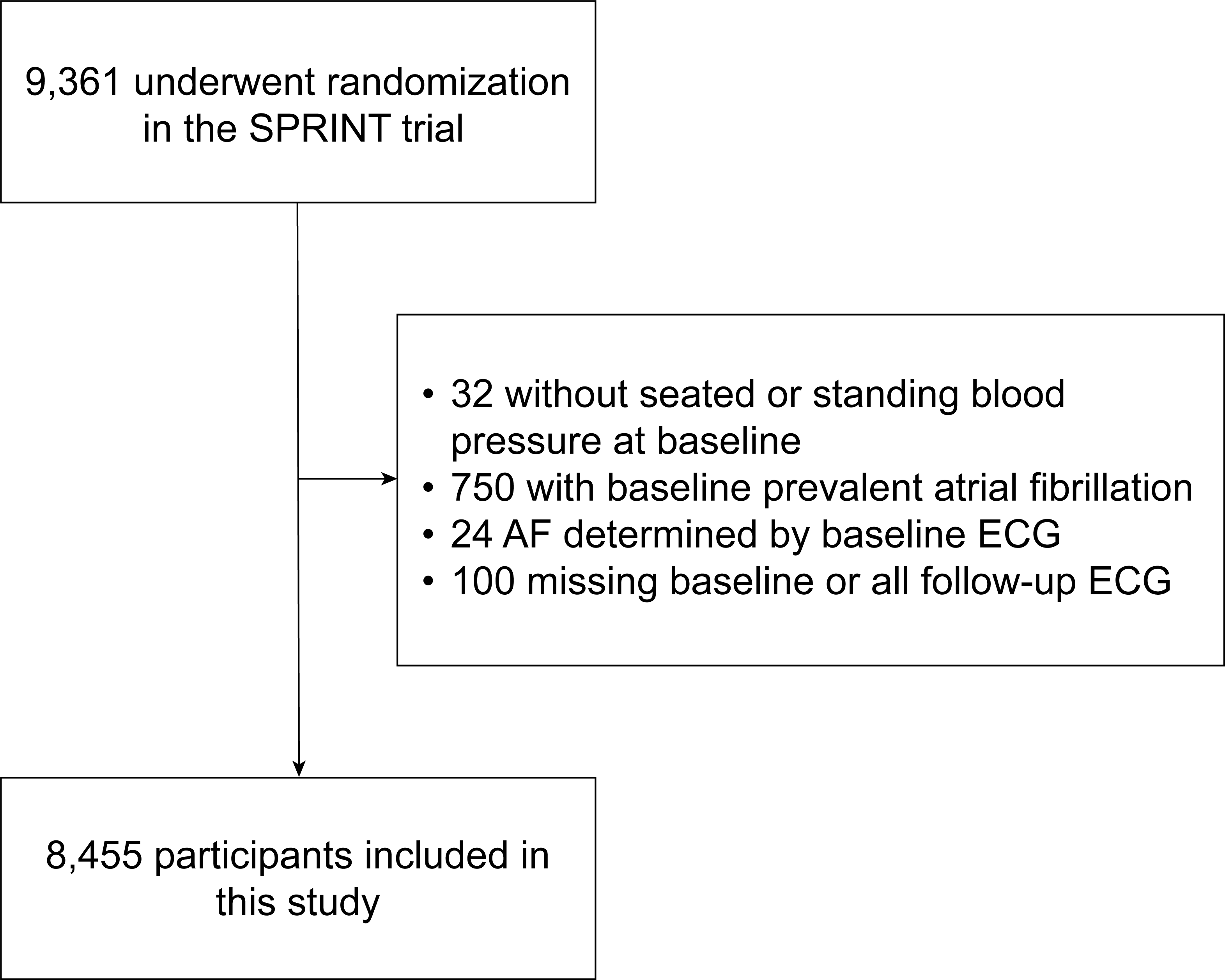


**Figure S1. Selection of study population**

SPRINT = systolic blood pressure intervention trial.

**
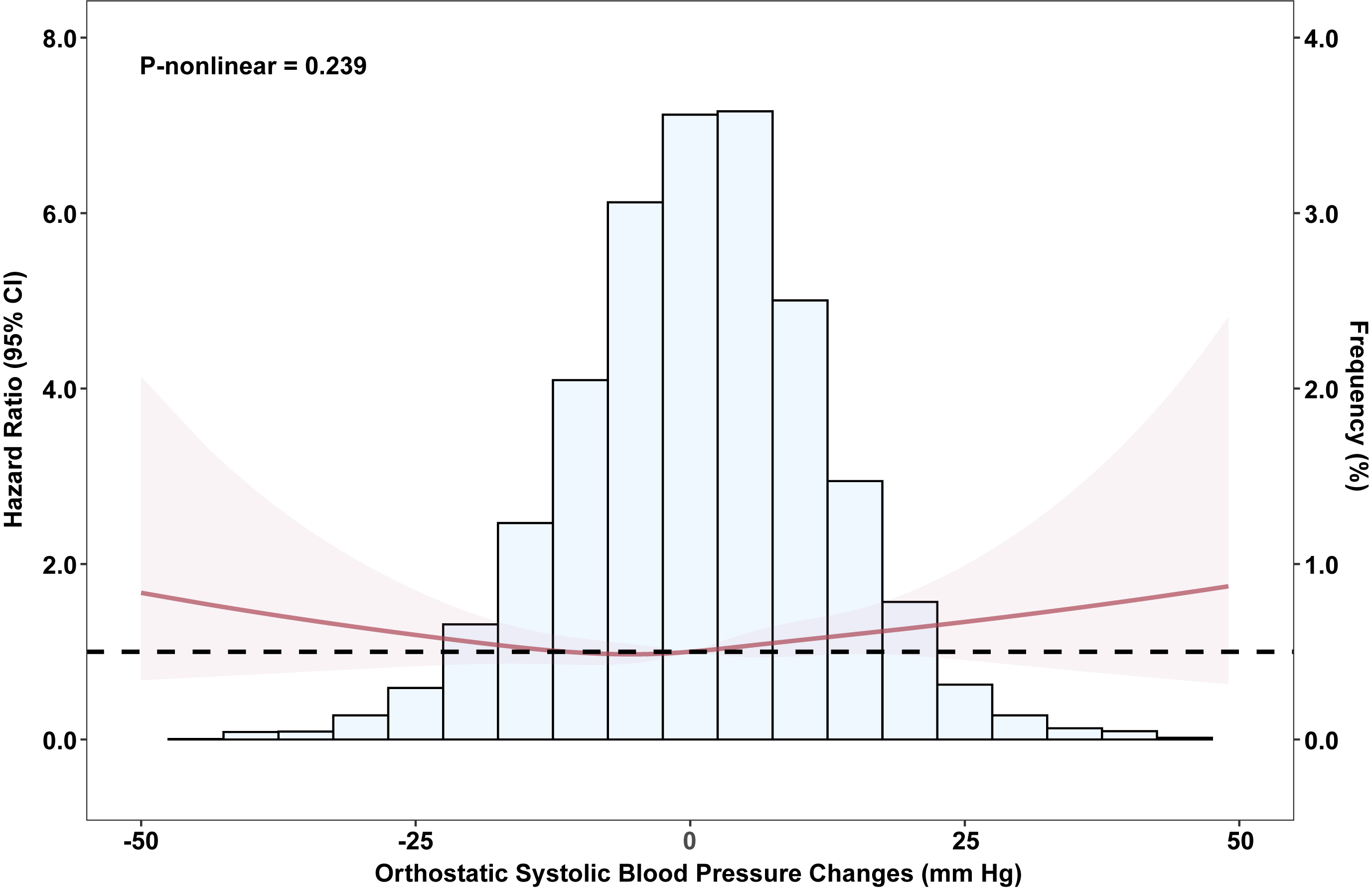
**

**Figure S2. Restricted cubic splines of orthostatic changes in systolic blood pressure to estimate atrial fibrillation development risk in the adjusted model.**

The orthostatic SBP changes were determined by subtracting the seated SBP from the standing SBP measurements.

The model was adjusted for age, sex, race, smoking, alcohol use, history of cardiovascular disease, history of chronic kidney disease, and body mass index.

SBP = systolic blood pressure.
